# A high-performance white-light-emitting-diodes based on nano-single crystal divanadates quantum dots

**DOI:** 10.1038/srep10460

**Published:** 2015-05-19

**Authors:** Weiqing Yang, Zhongli Liu, Jun Chen, Li Huang, Lei Zhang, Hong Pan, Bo Wu, Yuan Lin

**Affiliations:** 1 State Key Laboratory of Electronic Thin films and Integrated Devices, University of Electronic Science and Technology of China, Chengdu 610054, China; 2 Key Laboratory of Advanced Technologies of Materials (Ministry of Education), School of Materials Science and Engineering, Southwest Jiaotong University, Chengdu 610031, China; 3 College of Physics and Electronic Information, Luoyang Normal University, Luoyang 471022, China; 4 School of Materials Science and Engineering, Georgia Institute of Technology, Atlanta, GA 30332, USA; 5 National Synchrotron Radiation Laboratory and Hefei National Laboratory for Physical Sciences at Microscale, University of Science and Technology of China, Hefei, Anhui 230029, China

## Abstract

We report a high-performance phosphors-free white light-emitting-diodes (w-LEDs) using Ba_2_V_2_O_7_ or Sr_2_V_2_O_7_ quantum dots that directly heteroepitaxially grown on common quartz substrates by polymer assisted deposition (PAD). The quantum efficiency of quantum dots is as high as 95%. More importantly, electronic local functions, band structure and partial density of states have been firstly calculated to study the luminescent and heteroepitaxial growth mechanisms by the Ab-initio Simulation. Additionally, the glaring white light excited at a wavelength of 325 nm was experimentally observed, which unambiguously demonstrated that such quantum dots can be efficient w-LEDs for solid state lighting.

Usually, the conventionally structured white light-emitting-diodes (w-LEDs) rely on a three-ways coupling techniques of GaN-based blue LEDs, yellow-red phosphors and organic materials encapsulation^1^,^2^,^3^,^4^,^5^,^6^,^7^.But it often suffers from awkward predicaments, such as the relatively high fabrication cost of conventional yellow and red phosphors and comparatively low robustness due to peripherally organic encapsulating materials. These limitations greatly hampered the further commercially expanding of w-LEDs. With this regards, metavanadate phosphor films that directly fabricated on organic substrates at room temperature were innovatively reported for w-LEDs^8^. However, its low synthesis temperature regretfully led to the poor crystallinity of as-grown metavanadate films, which would largely sacrifice the luminescent properties. Additionally, the easily aging issue of organic substrates would also shorten the life time of the w-LEDs. Here, we demonstrate a novel high-performance phosphors-free w-LEDs, which is based on nano-single crystal Ba_2_V_2_O_7_ or Sr_2_V_2_O_7_ quantum dots (BVQD or SVQD) directly growing on common quartz substrates by polymer assisted deposition (PAD)^9^,^10^,^11^,^12^,^13^,^14^. Compared to the metavanadates-based phosphor films, the high quality BVQD or SVQD presents a broader band spectra in a range of 400 ~ 700 nm for w-LEDs with a higher quantum efficiency up to 95%. More importantly, for the first time, homogeneous nano-single crystal divanadates quantum dots have been successfully grown on common quartz substrates. Relying on the unique role of polymer bound metals, it truly brings a great breakthrough in the field of heteroepitaxial growth. Glaring white light excited at the wavelength of 325 nm was experimentally observed, which unambiguously demonstrated the capability of the nano-single crystal BVQD or SVQD as a novel phosphors-free w-LEDs model for solid state lighting. Moreover, the Ab-initio Simulation was employed for the first time to elucidate the luminescent and epitaxial growth mechanisms of BVQD or SVQD by calculating the electronic local functions (ELF), band structure and partial density of states (PDOS).

As schematically presented in Fig. 1a, the crystalline structure diagram shows that the desirable BVQD or SVQD heteroepitaxially grown on common SiO_2_ substrates. In a typical PAD process^9^,^10^,^11^,^12^,^13^,^14^, all the metal precursors, such as NH_4_VO_3_, Sr(NO_3_)_2_ and Ba(NO_3_)_2_, are first bounded to polymer as an ethylenediamineteraacetic acid (EDTA) complex to polyethyleneimine (PEI) in an aqueous solution. And then unbounded ions are filtered-off, leading to a homogeneous solution. For a desirable stoichiometry of BVQD or SVQD, inductively coupled plasma-atomic emission spectroscopy was used to determine the final concentration of each solution. As shown in Fig. 1b, these solutions were finally quantitatively mixed to obtain the desired stoichiometry, and then spin-coated onto the common quartz substrate. For the complete removal of the bound polymer, the thermal treatment was performed at 800 °C in oxygen for an hour. The heterogeneous nucleation of these bound metal ions was in process of becoming evenly distributed nano-crystal BVQD or SVQD on the surface of quartz (Fig. 1c). Subsequently, the second layer was spin-coated (Fig. 1d) and then annealed under the same condition (Fig. 1e). The BVQD or SVQD was growing layer by layer with the size of nano-single crystal gradually increases as the layer increases until the last ten layers (Fig. 1f).

As demonstrated in Figs. 2a,2b, scanning electron microscope (SEM) characterizes the surface microstructures of the nano-single crystal SVQD or BVQD on common quartz substrate, which indicates that the SVQD are homogenously distributed on the surface of quartz with an average diameter around 200 nm. The corresponding particle sizes distribution images of SVQD and BVQD are shown in Supplementary Fig. 1S. Additionally, the sectional SEM in Fig. 2c obviously shows that SVQD with a thickness of about 50 nm grows tightly on the common quartz surface, evidently indicating that PAD plays an important role in the common quartz-substrated heteroepitaxial growth of SVQD and BVQD. The BVQD shares a similar surface topography with SVQD, as SEM image shown in Supplementary Fig. 2S. Figs. 2d,2e are the transmission electron microscope (TEM) image of SVQD, in which, the size of SVQD is in good agreement with that from SEM results. Fig. 2f presented the atomic ratios characterized via EDX, which are well consistent with the nominal stoichiometry of SVQD. The FTIR spectrum of SVQD is shown in Fig. 2g. There are four characteristic peaks which correspond to the stretch vibrations of VO_4_ dimers^15^,^16^,^17^,^18^,^19^, namely, *v*_1_ = 581 cm^–1^, *v*_2_ = 748 cm^–1^, *v*_3_ = 813 cm^–1^ and *v*_4_ = 897 cm^–1^.

As shown in Fig. 2h, XRD patterns obviously demonstrate that BVQD grows along the special direction of <0*k*0> (*k* = 2, 4, 6) from PDF#039-1432 (please see the detailedly Supplementary materials), indicating an obtained high quality single crystal. Whereas the full width half maximum (FWHM) in <040> direction is only 0.25 (±0.01)°, which renders a big challenge for the heteroepitaxial growth of nano-single crystal on common non-crystalline substrate. SVQD also has the dramatically preferential growth direction along (0*k*0) (*k* = 2, 4, 6) except for the weak peak in the direction of <0–22>, suggesting a relatively fine nano-single crystal although its FWHM is up to 0.51(±0.02)°. In contrast, as demonstrated in Supplementary Fig. 3S, the related metavanadates thin film on organic substrate was very difficult to have such a good degree of crystallinity^8^.

Figure 3a shows the photoluminescence (PL) and excitation (PLE) spectra of SVQD and BVQD with ten layers when the emission wavelength and excited wavelength are *λ*_em_ = 550 nm and *λ*_ex_ = 325 nm, respectively. Each compound demonstrates a broad absorption band in PLE spectra in a range of 260 nm to 380 nm with a centre of 325 nm, and a broad-band emission spectrum spanning from 390 nm to 700 nm with a centre of 550 nm. The FWHM of emission band is as wide as about 180 nm. Moreover, the intensity of BVQD is slightly higher than that of SVQD due to the better degree of crystallinity (please see Fig. 1h). The *η* values of BVQD and SVQD measured at the wavelength of 325 nm at room temperature were respectively 95% and 93%, which are quite large among all the reported vanadate luminescent materials^8^,^15^,^16^,^17^. As shown in Fig. 3b, the color coordination of Commission Internationale de L’Eclairage (CIE) chromaticity diagram for BVQD and SVQD are CIE (0.361, 0.432) and CIE (0.342, 0.416), which are very close to pure white color CIE (0.33, 0.33).

The dependency of PL emission spectra on spin-coated layers for BVQD is presented in Fig. 3c. Since the quartz substrate shows no PL emission spectra, the observed PL emission light completely originates from BVQD. As the spin-coated layer increases, PL emission intensity gradually increases and finally reaches the stability. As shown in Fig. 3d, the peak intensities of PL emission spectra for both BVQD and SVQD obviously demonstrate the saturation tendency with the increase of deposition layers, which is ascribed to the saturation of crystalline growth.

The photographs in Figs. 3e,3f demonstrate the as-grown BVQD on common quartz, and the real-time bright white luminescence of BVQD excited by a 325 nm-wavelength ultraviolet light, which justifies the potential of nano-single crystal divanadate quantum dots as superior materials for new w-LEDs and other related solid state lighting devices.

In order to understand the growth and luminescent mechanism of BVQD on common quartz substrate, density functional theory (DFT) was performed for the nano-single crystal using the Vienna Ab-initio Simulation Package (VASP)^20^,^21^. The generalized gradient approximation (GGA)^22^ and the electron-ion interaction described by the projector augmented wave (PAW) scheme^23^,^24^. After full structure relaxation, parameters such as ELF, band structure and PDOS were systematically calculated. Figure 4a presents the exhaustively three-dimensional ELF of Ba_2_V_2_O_7_ <040>/SiO_2_<111>, in which, Ba_2_V_2_O_7_ and SiO_2_ interleave to each other with both their oxygens and the ELF of oxygens in SiO_2_ are obviously transformative due to the strong interfacial interaction. A corresponding interfacial section image was presented in Fig. 4b, which also clearly shows the interactions and theoretically describes the excellently heteroepitaxial growth of nano-single crystal on common non-crystalline substrate. Additionally, in Figs. 4c,4d, there are some electron transfer channels in left V-O bond not right V-O bond with an ELF value of 0.25, indicating that the luminescent is induced by one-electron charge-transfer transition from the oxygen 2*p* orbital to the vacant 3*d* orbital of V^5+^ in the tetrahedral VO_4_ with *T*_d_ symmetry^15^. Subsequently, getting an additional electron, the tetrahedral VO_4_ transforms the approximate *C*_3V_ symmetry, and in the meanwhile V^5+^ for 3*d*^0^ also changes into V^4+^ for 3*d*^1^. As demonstrated in Fig. 5, the band structure and PDOS of BVQD were also presented for a further quantificational illustration of the luminescent mechanism. The results indicate that the BVQD holds a direct band gap of approximately 2.25 eV at point *Γ* after geometrical optimization, which is very close to the peak value 2.259 eV (550 nm) in the emission spectra of BVQD. According to PDOS in Fig. 5, the top of valence band (VB) is close to 0 eV, which is dominated by O 2*p* orbitals. And the bottom of conduction band (CB) is mainly from V 3*d* orbitals.

In this regard, a possible mechanism of electron transition in BVQD system was proposed and schematically demonstrated in Fig. 6. The broad band emission for white light may consist of two parts. One part is the direct band gap transition from CB to VB, and that the transition probability of this part is often the most probable^15^,^25^,^26^,^27^. While the above experimental main peak value 2.259 eV (550 nm) is in good agreement with the theoretical direct band gap result 2.25 eV, evidently proving that this direct band gap transition should be the main origination of broad band emission. The other part may be the inter-band transition of V 3*d* orbital in CB^28^. Under the crystal-field interaction^29^,^30^, 3*d* orbital of V^4+^ ions with approximate *C*_3V_ symmetry was split into the different energy levels *Γ*_6_, *Γ*_4,5_, *Γ* ‘_4,5_, *Γ*_“6_ and *Γ* ‘_6_ with irreducible expression at the trigonal *C*_3v_ crystal field interaction as well as the spin-orbit interaction^15^. These inter-band transitions may constitute the other part of broad emission spectra. Thus, the above in-band and inter-band transitions of BVQD may coefficiently produce the broad band emission for white light.

In summary, we successfully demonstrated the high efficiency nano-single crystal Ba_2_V_2_O_7_ and Sr_2_V_2_O_7_ quantum dots directly heteroepitaxially on common substrates for phosphors-free white light emitting diodes by PAD. The high quality BVQD brings a broad band spectrum with a range of 400 ~ 700 nm and the corresponding quantum efficiency is up to 95%. Electronic local functions (ELF), band structure and partial density of states (PDOS) have evidently interpreted the luminescent and heteroepitaxial growth mechanisms of BVQD or SVQD. And the glaring white light excited at the wavelength of 325 nm was experimentally observed. Therefore, our demonstration paves the way towards a novel, simple and low cost phosphors-free w-LEDs model for solid state light.

## Methods

### Heteroepitaxially growing of BVQD or SVQD

To begin with 2.0 g NH_4_VO_3_ (Alfa Aesar), 3.0 g Ba(NO_3_)_2_ (Alfa Aesar) and 3.0 g Sr(NO_3_)_2_ (Alfa Aesar) were dissolved in the same 50 mL distilled water including previously dissolved 4.0 g polyethyleneimine (PEI, average Mn ≈ 60000, Mw ≈ 750000) (Sigma Aldrich) and 4.0 g ethylene diamine tetraacetic acid (EDTA) (Sigma Aldrich) to form a homogeneous polymer precursor solution. And then, the solution was purified in an Amicon filtration (Amicon 8050) unit and concentrated to get a precursor solution. An Inductively Coupled Plasma Optical Emission Spectrometer (ICP-OES) was used to measure the concentrations for metal ions of each precursor solution. According to the corresponding mol ratio of BVQD or SVQD, two precursor solutions were uniformly mixed into the final precursor solution, which was spin-coated on the common quartz substrates at a speed of 3000 r.p.m. The precursor films were thermally treated at 800 °C for an hour with both the temperature rise and fall ratios of 2 °C per minute, and then came into being BVQD or SVQD. Spin-coating and thermal treating were repeatedly performed to obtain the BVQD or SVQD with desired size and thickness.

### Nano-single crystal characterization

**Figure 3:**
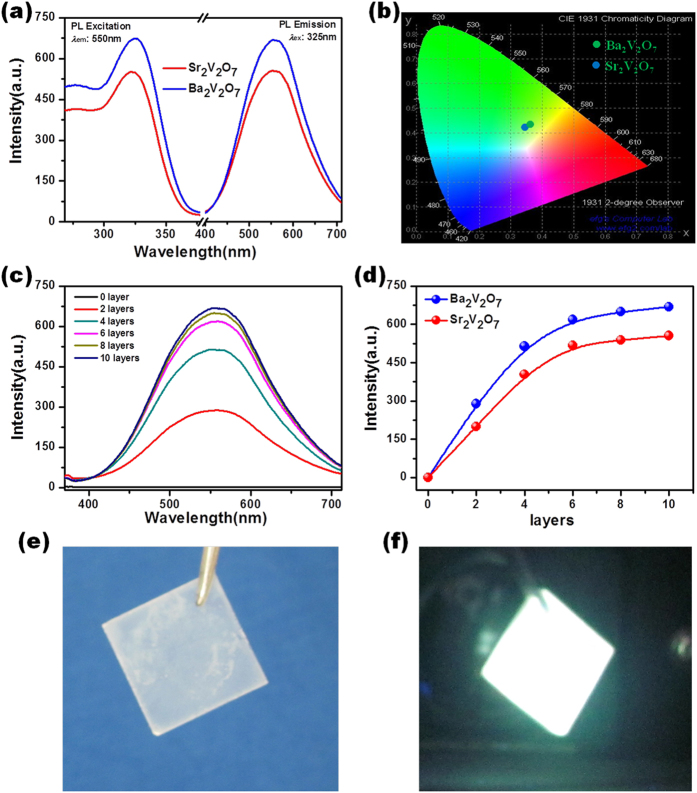
The luminescent properties of SVQD and BVQD. **a**, PLE and PL spectra for SVQD and BVQD. The excitation spectra are monitored at 550 nm, and the emission spectra are obtained by excitation at 325 nm. **b**, CIE chromaticity diagram showing the color coordination for SVQD and BVQD. **c**, PL emission spectra for BVQD with various layers by excitation at 325 nm. **d**, The dependence of PL intensity on the numbers of SVQD or BVQD layers. **e**, **f**, A photograph of BVQD on common quartz substrate and its real-time emission performance with an excitation at 325 nm.

The X’Pert Pro MPD (Philips, Holland) X-ray diffractometer with Cu Ka1 radiation (*λ* = 0.154 nm) was applied to determine the crystal structures of BVQD or SVQD. The morphology, compositions and crystallography of as-grown quantum dots were examined by SEM (Hitachi S4800, Japan), EDS (Hitachi S4800, Japan) and TEM (Tecnai G2 F20 S-Twin, USA). The FTIR spectra for sample was measured by, IRPrestige-21 (Shimadzu, Japan). The luminescent properties and corresponding *η* values of as-grown materials were measured by FL3-TCSPC (Horiba Jobin Yvon, England) equipped with an integrated sphere with a size of 6 inches. All the luminescent measurements were finished at the atmosphere of room temperature, and the solid state BVQD and SVQD on quartz substrates (please see the photograph of Fig. 3e) were directly measured.

## Author Contributions

W.Y., Z.L. and Y.L. wrote the main manuscript. Z.L. finished all the calculations. J.Chen revised the main manuscript. W.Y., L.H., L.Z., H.P. and B.W. finished all the experiments. All authors reviewed the manuscript.

## Additional Information

**How to cite this article**: Yang, W. *et al*. A high-performance white-light-emitting-diodes based on nano-single crystal divanadates quantum dots. *Sci. Rep.* **5**, 10460; doi: 10.1038/srep10460 (2015).

## Supplementary Material

Supporting Information

## Figures and Tables

**Figure 1:**
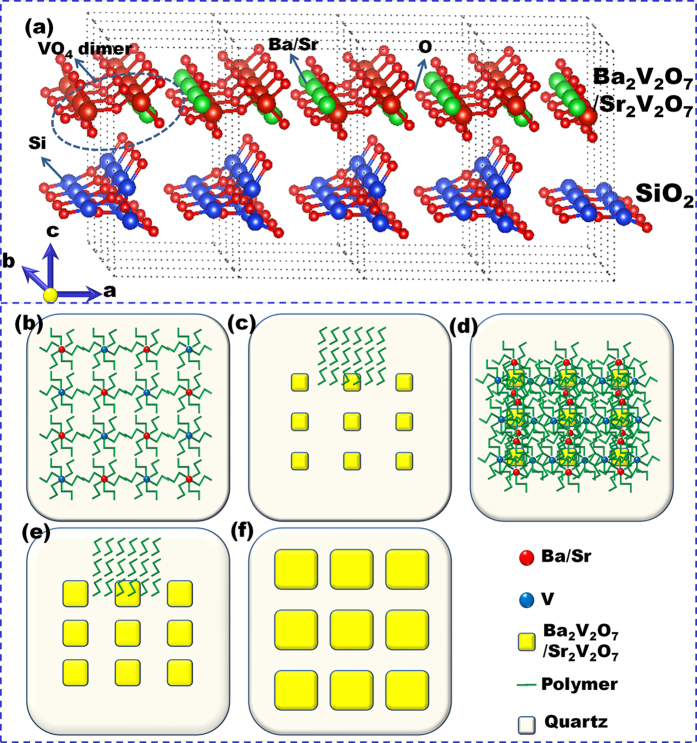
A schematical illustration of the growth process of nano-single crystal SVQD or BVQD on common quartz substrate. **a**, The molecular structure diagram. **b-f**, A whole growth process sketch of SVQD or BVQD (the metal precursors evenly bounded by polymer were spin-coated onto the common quartz substrate and then a thermal treatment was performed for the complete remova the bound polymer. The BVQD or SVQD was growing layer by layer with the same conditions until the last ten layers.

**Figure 2:**
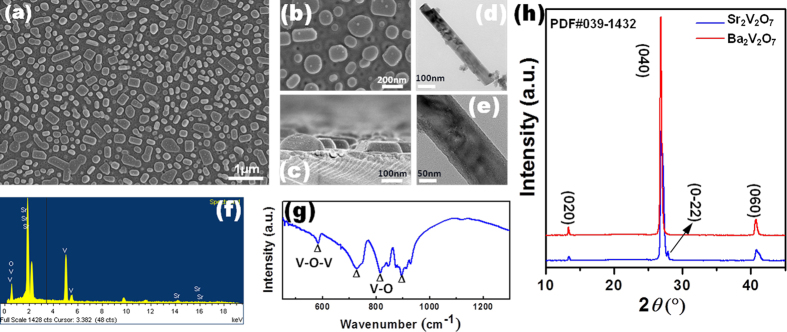
Structural and surface morphological characterization of SVQD or BVQD. **a**, SEM image. **b**, An enlarged view of the SEM image. **c**, A sectional SEM image of SVQD. **d, e**, TEM and an enlarged view of TEM image for SVQD. **f**, **g**, EDS and FTIR spectrum of as-grown SVQD. **h**, XRD patterns of BVQD and SVQD.

**Figure 4:**
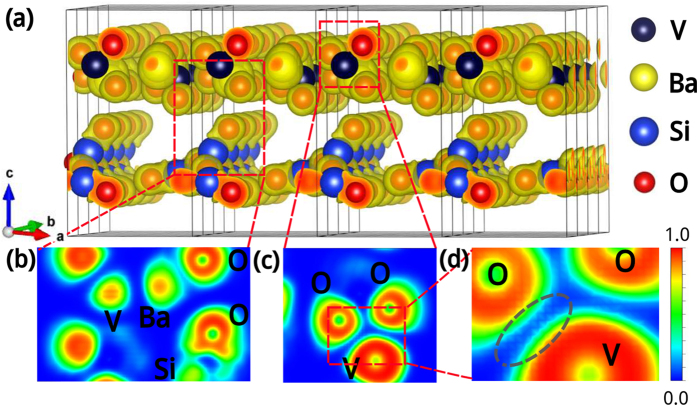
The electronic local functions (ELF) of BVQD/quartz. **a**, Three-dimensional ELF of Ba_2_V_2_O_7_ <040>/SiO_2_<111>. **b**, **c**, its corresponding two-dimensional interfacial sectional images. **d**, the enlarged view of **c**.

**Figure 5:**
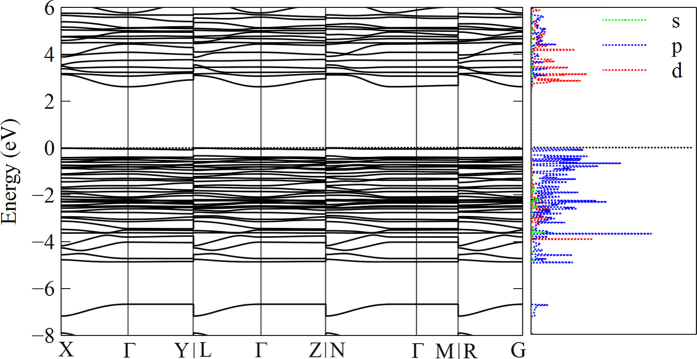
Electronic structure characterization of Ba_2_V_2_O_7_ <040>/SiO_2_<111>. The band structure (left) and partial density of states (PDOS) (right) of BVQD.

**Figure 6:**
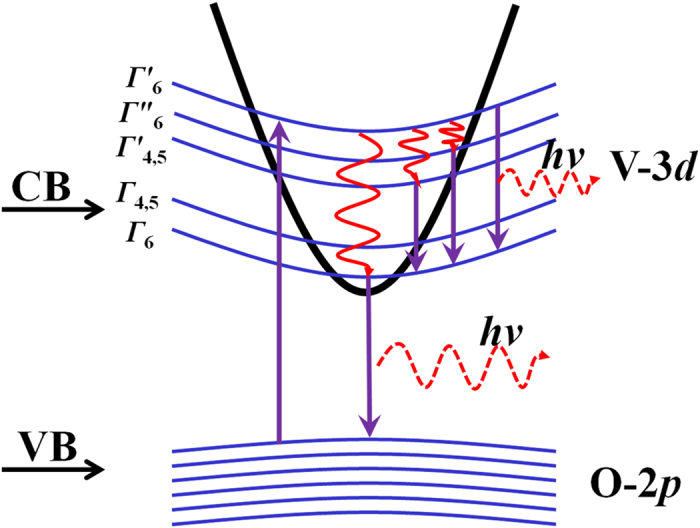
A schematical illustration of the luminescent mechanism of BVQD or SVQD.
