# Supplementary material for: A high-performance white-light-emitting-diodes based on nano-single crystal divanadates quantum dots
Source: Sci Rep. 2015 May 19;5:10460. doi: 10.1038/srep10460 (PMC4437379; doi:10.1038/srep10460)
Supplement: Supporting Information [file srep10460-s1.pdf]

Supplementary Information

A high-performance white-light-emitting-diodes based on nano-single crystal divanadates quantum dots

Weiqing Yang, [1,2,#] Zhongli Liu,[3,#] Jun Chen, [4] Li Huang, [5] Lei Zhang, [2] Hong Pan, [2] Bo Wu,[1] Yuan Lin [1,*]

[1]State Key Laboratory of Electronic Thin films and Integrated Devices, University of Electronic Science and Technology of China, Chengdu 610054, China

[2]Key Laboratory of Advanced Technologies of Materials (Ministry of Education), School of Materials Science and Engineering, Southwest Jiaotong University, Chengdu 610031, China

[3]College of Physics and Electronic Information, Luoyang Normal University, Luoyang 471022, China

[4]School of Materials Science and Engineering, Georgia Institute of Technology, Atlanta, GA 30332, USA

[5]National Synchrotron Radiation Laboratory and Hefei National Laboratory for Physical Sciences at Microscale, University of Science and Technology of China, Hefei, Anhui 230029, China

[#] Authors contributed equally to this work.

[*] Corrsponding authors. Tel.:+86 28 87600415
E-mail address: wqyang@home.swjtu.edu.cn and linyuan@uestc.edu.cn

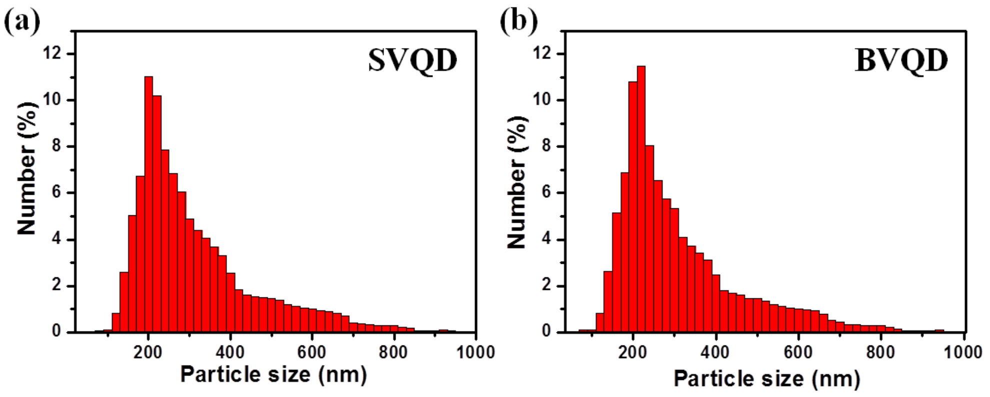

Fig. S1. The particle sizes distribution images of (a) SVQD and (b) BVQD.

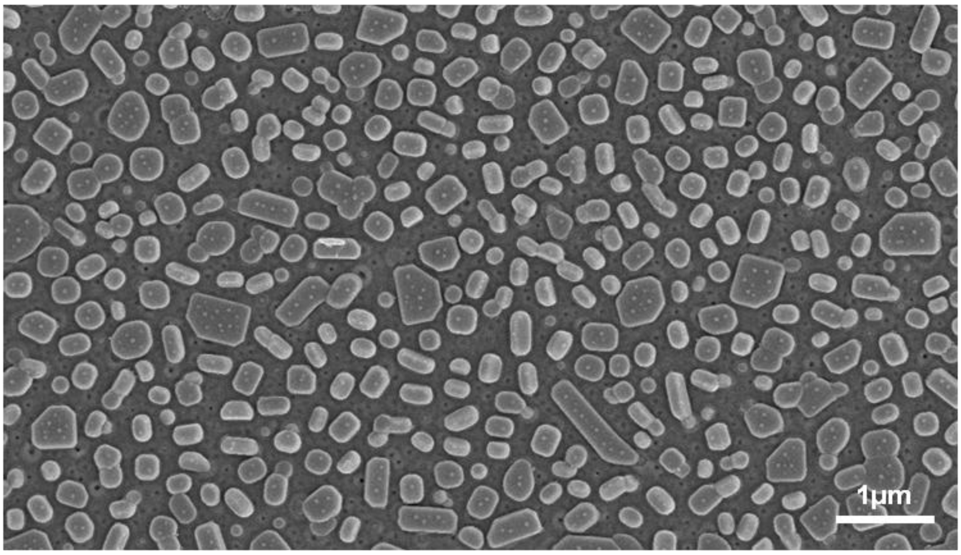

Fig. S2 The SEM image of BVQD.

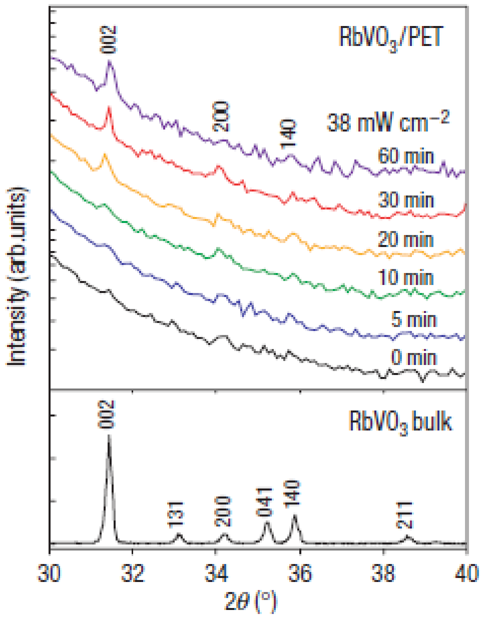

Fig. S3 The replicated image of XRD pattern for metavanadates phosphors film on organic substrate from Ref.8

PDF#39-1432:

QM=Star/Calculated; d=Other/Unknown; I=(Unknown)

Barium Vanadium Oxide

Ba2V2O7

Radiation=CuKa1 Lambda=1.5406 Filter=

Calibration= d-Cutoff= I/Ic(RIR)=

Ref= Level-1 PDF

Triclinic, P-1(2) Z=4 mp=

Cell=7.324x13.564x7.322<92.65x89.76x99.47> Pearson=

Density(c)=4.527 Density(m)= Mwt= Vol=

Ref= Ibid.

Strong Line: 3.34/X 3.49/8 3.24/8 3.61/6 3.64/6 2.89/5 3.27/5 3.66/4

d(A)	I(f)	I(v)	h	k	l	n^2	2-Theta	Theta	1/(2d)	2pi/d
6.678	8.0	4.0	0	2	0		13.247	6.623	0.0749	0.9408
5.966	7.0	4.0	1	1	0		14.837	7.418	0.0838	1.0532
5.149	18.0	12.0	-1	0	1		17.207	8.603	0.0971	1.2202
4.677	11.0	8.0	-1	-1	1		18.957	9.478	0.1069	1.3432
4.107	7.0	6.0	-1	3	0		21.616	10.808	0.1217	1.5296
3.923	14.0	12.0	-1	-2	1		22.646	11.323	0.1274	1.6016
3.802	8.0	7.0	1	2	1		23.376	11.688	0.1315	1.6525
3.729	7.0	6.0	0	3	1		23.836	11.918	0.1341	1.6845
3.655	42.0	38.0	0	0	2		24.329	12.165	0.1368	1.7189
3.642	60.0	55.0	-2	1	0		24.418	12.209	0.1373	1.7251
3.612	60.0	55.0	2	0	0		24.623	12.312	0.1384	1.7393
3.570	19.0	18.0	0	-1	2		24.915	12.458	0.1400	1.7596
3.485	84.0	81.0	0	1	2		25.534	12.767	0.1434	1.8026
3.423	31.0	30.0	-2	2	0		26.006	13.003	0.1461	1.8354
3.341	100.0	100.0	0	4	0		26.656	13.328	0.1496	1.8804
3.273	45.0	46.0	0	-2	2		27.221	13.611	0.1528	1.9195
3.236	77.0	80.0	-1	-3	1		27.539	13.770	0.1545	1.9415
3.199	24.0	25.0	-1	1	2		27.862	13.931	0.1563	1.9638
3.140	20.0	21.0	1	3	1		28.399	14.200	0.1592	2.0009
3.077	42.0	46.0	-2	2	1		28.986	14.493	0.1625	2.0414
3.029	8.0	9.0	2	1	1		29.457	14.729	0.1650	2.0739
3.015	18.0	20.0	1	-4	1		29.597	14.799	0.1658	2.0835
2.978	23.0	26.0	2	2	0		29.979	14.990	0.1679	2.1098
2.893	45.0	52.0	0	-3	2		30.878	15.439	0.1728	2.1715
2.857	10.0	12.0	1	4	0		31.274	15.637	0.1750	2.1987
2.852	5.0	6.0	2	-3	1		31.337	15.669	0.1753	2.2030
2.785	15.0	18.0	1	-3	2		32.109	16.055	0.1795	2.2558
2.681	14.0	17.0	-2	4	0		33.387	16.694	0.1865	2.3431
2.625	10.0	13.0	1	4	1		34.119	17.060	0.1904	2.3930
2.604	8.0	10.0	2	3	0		34.401	17.201	0.1920	2.4122
2.592	20.0	26.0	2	-1	2		34.573	17.287	0.1929	2.4239
2.573	22.0	29.0	-2	0	2		34.829	17.415	0.1943	2.4413
2.571	21.0	27.0	-2	1	2		34.867	17.434	0.1945	2.4438
2.548	9.0	12.0	0	-5	1		35.189	17.595	0.1962	2.4657
2.526	7.0	9.0	0	-4	2		35.499	17.750	0.1979	2.4867
2.491	7.0	9.0	-2	4	1		36.019	18.010	0.2007	2.5220
2.480	15.0	20.0	1	-4	2		36.190	18.095	0.2016	2.5335
2.460	18.0	24.0	-1	5	1		36.493	18.247	0.2032	2.5540
2.438	7.0	10.0	0	0	3		36.825	18.413	0.2050	2.5764
2.410	3.0	4.0	0	4	2		37.280	18.640	0.2075	2.6072
2.341	7.0	10.0	-2	5	0		38.421	19.211	0.2136	2.6840
2.317	14.0	20.0	3	-1	1		38.827	19.414	0.2158	2.7113
2.309	20.0	29.0	1	-1	3		38.959	19.479	0.2165	2.7201
2.303	23.0	33.0	3	1	0		39.072	19.536	0.2171	2.7277
2.282	27.0	40.0	2	2	2		39.439	19.720	0.2190	2.7523
2.272	30.0	44.0	2	4	0		39.620	19.810	0.2200	2.7644
2.255	21.0	31.0	0	2	3		39.931	19.966	0.2216	2.7852
2.248	15.0	22.0	1	-2	3		40.065	20.032	0.2224	2.7942
2.236	10.0	15.0	1	5	1		40.289	20.144	0.2235	2.8092
2.228	13.0	19.0	0	6	0		40.452	20.226	0.2244	2.8201
2.208	24.0	36.0	1	4	2		40.828	20.414	0.2264	2.8452
2.193	20.0	30.0	1	-5	2		41.119	20.559	0.2280	2.8646
2.180	11.0	17.0	-1	-2	3		41.377	20.689	0.2293	2.8818
2.165	15.0	23.0	-3	3	1		41.667	20.834	0.2309	2.9011
2.158	20.0	31.0	0	-6	1		41.813	20.906	0.2316	2.9108
2.150	20.0	31.0	2	4	1		41.980	20.990	0.2325	2.9219
2.134	8.0	13.0	1	-3	3		42.303	21.152	0.2342	2.9434

2.128	13.0	20.0	-2	4	2	42.435	21.217	0.2349	2.9521
2.110	19.0	30.0	-1	6	1	42.821	21.410	0.2370	2.9777
2.105	14.0	22.0	0	6	1	42.927	21.464	0.2375	2.9847
2.096	10.0	16.0	0	3	3	43.108	21.554	0.2385	2.9967
2.092	11.0	18.0	2	3	2	43.194	21.597	0.2389	3.0024
2.056	8.0	13.0	3	2	1	43.985	21.993	0.2431	3.0547
2.052	7.0	11.0	-2	6	0	44.084	22.042	0.2436	3.0612
2.045	7.0	11.0	-1	-3	3	44.245	22.123	0.2445	3.0719
2.033	6.0	10.0	2	-1	3	44.525	22.263	0.2459	3.0903
2.018	11.0	18.0	-2	1	3	44.876	22.438	0.2478	3.1134
2.015	15.0	25.0	-3	0	2	44.942	22.471	0.2481	3.1177
2.006	10.0	17.0	2	-5	2	45.162	22.581	0.2493	3.1322
1.991	12.0	20.0	-3	2	2	45.507	22.753	0.2511	3.1548
1.985	12.0	20.0	-2	-1	3	45.647	22.824	0.2518	3.1641
1.957	21.0	36.0	2	1	3	46.343	23.171	0.2554	3.2096
1.952	22.0	38.0	3	-3	2	46.463	23.232	0.2560	3.2175

Acknowledgements

This work is supported by the National Natural Science Foundation of China (Nos. 51202023 and 11104127), China Postdoctoral Science Foundation (No. 2013T60845 and 2012M511917) and the Fundamental Research Funds for the Central Universities (A0920502051408-10).

Author contributions statements

W. Q. Yang, Z. L. Liu and Y. Lin wrote the main manuscript. Z. L. Liu finished all the calculations. J. Chen revised the main manuscript. W. Q. Yang, L. Huang, L. Zhang, H. Pan and B. Wu finished all the experiments. All authors reviewed the manuscript.

Additional information

Competing financial interests: The authors declare no competing financial interests.
